# Two types of functionally distinct Ca^2+^ stores in hippocampal neurons

**DOI:** 10.1038/s41467-019-11207-8

**Published:** 2019-07-19

**Authors:** Hsing-Jung Chen-Engerer, Jana Hartmann, Rosa Maria Karl, Jun Yang, Stefan Feske, Arthur Konnerth

**Affiliations:** 1 Institute of Neuroscience, Technical University of Munich, Biedersteiner Str. 29, 80802 Munich, Germany; 2 Munich Cluster for Systems Neurology (SyNergy) and Center for Integrated Protein Sciences (CIPSM), Biedersteiner Str. 29, 80802 Munich, Germany; 3 Department of Pathology, School of Medicine, New York University, New York, NY 10003 USA

**Keywords:** Cell biology, Cellular neuroscience

## Abstract

It is widely assumed that inositol trisphosphate (IP_3_) and ryanodine (Ry) receptors share the same Ca^2+^ pool in central mammalian neurons. We now demonstrate that in hippocampal CA1 pyramidal neurons IP_3_- and Ry-receptors are associated with two functionally distinct intracellular Ca^2+^ stores, respectively. While the IP_3_-sensitive Ca^2+^ store refilling requires Orai2 channels, Ry-sensitive Ca^2+^ store refilling involves voltage-gated Ca^2+^ channels (VGCCs). Our findings have direct implications for the understanding of function and plasticity in these central mammalian neurons.

## Abstract (web summary)

It is generally assumed, from previous studies in cerebellar Purkinje neurons, that IP_3_ and Ry receptors share the same intracellular Ca^2+^ pool. In this study, the authors demonstrate the existence of two functionally distinct Ca^2+^ stores in mouse CA1 pyramidal neurons by showing that refilling of IP_3_-sensitive stores relies on Orai2 channels, whereas Ry-sensitive stores involve VGCCs for spontaneous store refilling

## Introduction

Ca^2+^ ions are ubiquitous and versatile signaling molecules that control a plethora of cellular processes in virtually all biological organisms. In mammalian neurons, transient changes in the cytosolic Ca^2+^ concentration control, for example, transmitter release, the induction of activity-dependent synaptic plasticity, and gene expression^1^. A major source of such neuronal Ca^2+^ signals is Ca^2+^ entry from the extracellular space through Ca^2+^-permeable channels, mostly through voltage-gated Ca^2+^ channels (VGCCs) in the plasma membrane. Another essential source is the intracellular release of Ca^2+^ ions from endoplasmic reticulum (ER) Ca^2+^ stores, which is mediated by two types of receptors in the ER membrane, namely, inositoltrisphosphate (IP_3_) and ryanodine (Ry) receptors^2^. Both IP_3_ and Ry receptors are abundantly expressed in most central mammalian neurons, including CA1 pyramidal neurons (PNs) and cerebellar Purkinje cells^3^. Although the experimental evidence is rather scarce, it is widely assumed that IP_3_ and Ry receptors share the same intracellular Ca^2+^ pool (e.g. ref. ^4^). However, the intracellular distribution of IP_3_ and Ry receptors is not homogenous. For example, in Purkinje cells, IP_3_ receptors are enriched in dendritic spines, while Ry receptors are more abundant in dendritic shafts^5^. An opposite expression pattern was observed in CA1 PNs^6,7^. Interestingly, although there is not much support of a strict receptor co-localization, it is believed that IP_3_ and Ry receptors have largely overlapping cellular functions^8^.

The homeostasis of the intraluminal Ca^2+^ concentration in the ER is maintained by Ca^2+^ entry from the extracellular space^9^. For example, in CA1 PNs, the refilling of Ry-sensitive Ca^2+^ stores was suggested to be mediated by VGCCs^10^. However, most central neurons abundantly express STIM (stromal interaction molecules) and Orai^3,11,12^, which in non-excitable cells form a signaling complex that accounts for the refilling in ER Ca^2+^^13–17^. The roles of STIM and Orai in central mammalian neurons are barely understood. Recent studies determined a neuronal cell type-specific expression of STIM homologs, with, for example, STIM1 predominantly present in Purkinje neurons^11^ and STIM2 predominant in pyramidal^3,12^. In these neurons, STIM1 and STIM2 are determinants of ER Ca^2+^^11,12^. However, the identity and the functions of the corresponding Orai channels is unknown. Here we set out to investigate the specific roles of Orai1 and Orai2 channels for the function(s) of IP_3_ and Ry receptor-sensitive ER Ca^2+^ stores in CA1 PNs by using Orai-deficient mouse lines.

## Results

**Fig. 1:**
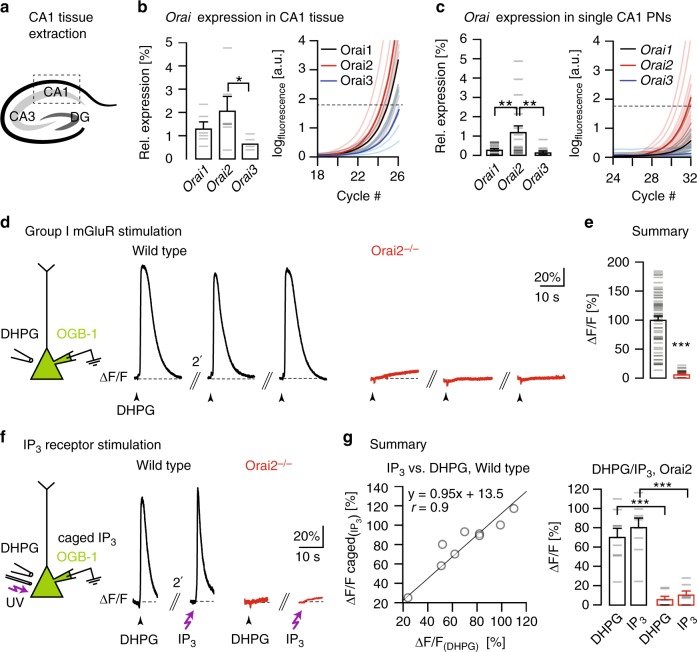
Orai2 is required for IP_3_R-dependent Ca^2+^ release from internal stores. **a** Schematic depiction of the hippocampus with the three regions CA1, CA3, and the dentate gyrus (DG). CA1 tissue was extracted for quantitative reverse transcription-PCR. **b** Result of Orai PCR analysis in the mouse hippocampus (P18). Left: Mean expression levels of the three Orai homologs relative to the housekeeping gene *Gapdh* (*n* = 6 mice, *p* = 0.024 (analysis of variance)). Gray dashes represent individual values in this and other figures. Right: Real-time monitoring of the fluorescence emission of SYBR Green I during the PCR amplification of Orai1-3 cDNA. Dashed line: noise band. **c** Analogous PCR analysis in single CA1 pyramidal neurons (PNs) harvested from acute hippocampal slices. Orai1: *n* = 21, Orai2: *n* = 19, Orai3: *n* = 15 cells; *p* = 0.002 (Orai1–Orai2), *p* = 0.001 (Orai2–Orai1). **d** Left: DHPG was pressure-applied to somata of whole-cell patch-clamped CA1 PNs filled through the patch pipette with OGB-1. Black traces: Ca^2+^ transients in CA1 PNs in a wild-type mouse (left) in response to repeated applications of DHPG (500 µM, 200 ms) at 2-min intervals. Red traces: Analogous experiments in an Orai2−/− mouse. **e** Mean amplitudes of DHPG-evoked Ca^2+^ transients in wild-type and Orai2−/− mice (*n* = 62 and 33 cells, respectively; *p* = 9.84 × 10^−24^). **f** Left: Both ultraviolet light pulses and local DHPG puffs were applied to somata of whole-cell patch-clamped CA1 PNs filled with OGB-1 and NPE (“caged”)-IP_3_ through the patch pipette. Black traces: Fluorescence transients resulting from photolysis of caged IP_3_ (left) and local DHPG puff application (right) in the same cell in a wild-type mouse. Red traces: Analogous experiment in an Orai2−/− mouse. **g** Left: Correlation plot of relative fluorescence changes in response to IP_3_-uncaging (*y* axis) against relative fluorescence changes in response to DHPG applications (*x* axis) in the same cells in the wild type. Right: Mean amplitudes of relative fluorescence changes in response to both treatments in **c** in both genotypes (*n* = 9 cells in wild type and 7 cells in Orai2−/− mice, *p* = 1.74 × 10^−4^, *p* = 3.5 × 10^−4^). * - significant (*p* < 0.05), ** -  very significant (*p* < 0.01), *** - highly significant (*p* < 0.001)

We first analyzed the expression of Orai Ca^2+^ channels, known to regulate Ca^2+^ store refilling in non-excitable cells^15,16^, in mouse CA1 PNs. We determined mRNA expression of all three *Orai* genes (*Orai1–3*) in the CA1 region of the mouse hippocampus (Fig. 1a–c) by quantitative real-time polymerase chain reaction (qPCR)^18,19^ and reliably detected transcripts for all three *Orai* homologs. The mean expression levels relative to the housekeeping gene *Gapdh* were 1.3 ± 0.3% for *Orai1*, 2 ± 0.6% for *Orai2*, and 0.6 ± 0.1% for *Orai3* (*n* = 6 mice for all; Fig. 1b). The analogous analysis in 6 mice of a mouse line with a targeted deletion of the *Orai2* gene (Orai2−/−^20^) yielded similar relative expression for *Orai1* (1.0 ± 0.1%) and *Orai3* (0.5 ± 0.9%) and, as expected, the absence of *Orai2* mRNA (Supplementary Fig. 1a). Furthermore, we performed a quantitative reverse transcription-PCR (RT-PCR) analysis for the *Orai* genes on the level of single CA1 PNs^18^. The mean expression level of *Orai2* in single neurons, in contrast to the CA1 tissue, was higher than that of *Orai1* and *Orai3* (0.3% ± 0.05% for *Orai1*, *n* *=* 21 cells; 1.2 ± 0.3% for *Orai2*, *n* *=* 19 cells; 0.1 ± 0.05% for Orai3, *n* = 15 cells; Fig. 1c). For immunohistochemical staining of Orai2 expression patterns in CA1, we made use of the fact that in the Orai2−/− mice the protein-coding exons of *Orai2* were replaced with a *LacZ* reporter. Accordingly, in wild-type (WT) mice, β-gal (5-bromo-4-chloro-3-indolyl β-d-galactopyranoside) staining yielded a weak and unspecific staining (Supplementary Fig. 1b, left), whereas a bright signal for β-gal was found in the somatodendritic compartment of CA1 PNs in Orai2−/− mice, confirming both the deletion of the *Orai2* gene in the mutant and its expression in the WT mouse lines, respectively (Supplementary Fig. 1b, right).

We analyzed the functional status of IP_3_-sensitive Ca^2+^ stores by locally applying to CA1 PNs 3,5-dihydroxyphenylglycine (DHPG), an agonist of group I metabotropic glutamate receptors (mGluR1 and mGluR5), known to be abundantly expressed^3^ and of major functional importance in these neurons^21^. Fig. 1d, e demonstrates that DHPG application reliably evoked Ca^2+^ release signals from stores (see Supplementary Fig. 2a–d) in WT mice (left) but not in Orai2-deficient (Orai2−/−) mice (right) (see also control experiments in Supplementary Fig. 3). In contrast, the deletion of Orai1 had no detectable impact on the Ca^2+^ release signals from stores (see Supplementary Fig. 4a–c). Thus Orai channels in CA1 PNs differ functionally from those expressed in electrically non-excitable immune cells, where the deletion of Orai1 reduces SOCE while deletion of Orai2 increases SOCE^20^. Next, we explored the reason for the absence of DHPG-induced Ca^2+^ transients by testing the effect of photolysis of NPE-caged IP_3_ (myo-inositol-1,4,5-triphosphate, P_(4,5)_-1-(2-nitrophenyl)ethyl ester). We combined recordings of DHPG applications and IP_3_ uncaging in the same CA1 PNs and found similar responsiveness and unresponsiveness patterns in WT and Orai2−/− mice, respectively (Fig. 1f, g). Together, these results established a requirement of Orai2 for the normal function of IP_3_-sensitive Ca^2+^ stores in CA1 PNs.

**Fig. 2:**
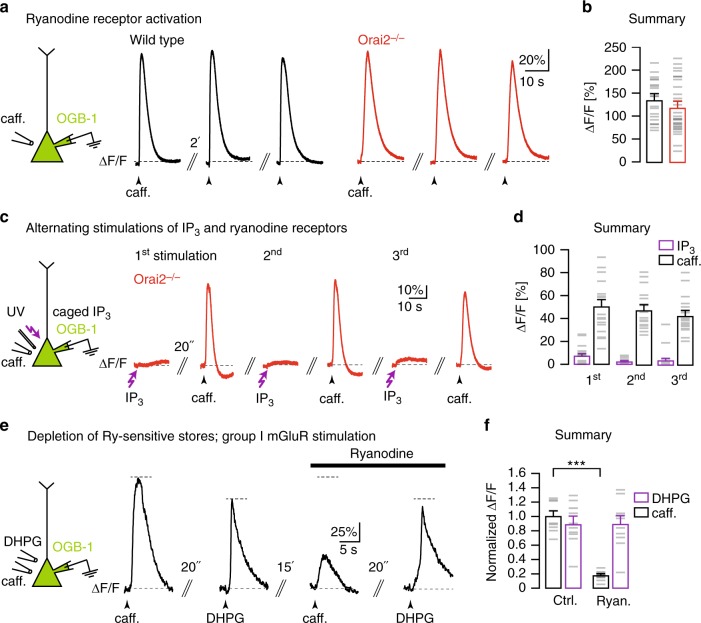
Orai2 is not required for RyR-dependent Ca^2+^ release from internal stores. **a** Left: Caffeine was locally applied to the somata of whole-cell patch-clamped CA1 pyramidal neurons (PNs) filled with OGB-1 through the patch pipette. Black traces: Fluorescence transients in response to three consecutive caffeine applications with an interval of 2 min in a wild-type mouse. Red traces: Analogous experiment in an Orai2-deficient mouse. **b** Summary of the experiments in **a**. Bar graph shows the mean amplitudes of relative fluorescence changes resulting from the first caffeine application in each cell (*n* = 20 cells in wild-type and *n* = 28 cells in Orai2−/− mice, *p* = 0.268). **c** Left: Both caffeine and ultraviolet pulses were applied to somata of whole-cell patch-clamped CA1 PNs filled through the patch pipette with NPE (caged)-IP_3_ and OGB-1. Red traces: Fluorescence transients resulting from three cycles of alternating somatic IP_3_ uncaging and caffeine applications in an Orai2−/− mouse. **d** Summary of experiments in **c**. Bar graph showing the mean amplitudes of responses to caffeine (white) and IP_3_ uncaging (purple; *n* = 18 cells). **e** Left: Both caffeine and 3,5-dihydroxyphenylglycine (DHPG) were locally applied to the somata of whole-cell patch-clamped CA1 PNs filled with OGB-1 through the patch pipette. Traces: Fluorescence transients in response to somatic caffeine and DHPG applications with the indicated time intervals in control artificial cerebrospinal fluid (left traces) and in the presence of 10 µM ryanodine (right traces). **f** Summary bar graph showing mean amplitudes of relative fluorescence changes for all experiments (*n* = 10 cells, *p* = 7.89 × 10^−9^) in **e**. *** - extremely significant (*p* < 0.0001)

How about Ry-sensitive intracellular Ca^2+^ stores? Earlier work had established that, in CA1 PNs, RyRs can be effectively activated by local application of caffeine^10^. We confirmed this effect in WT mice (Fig. 2a, left). Surprisingly, however, caffeine-induced Ca^2+^ release from stores (Fig. 2e, f) was unaltered both in Orai2−/− mice (Fig. 2a, right, Fig. 2b) and in Orai1-deficient mice (Supplementary Fig. 4d, e). By using IP_3_ uncaging in combination with caffeine application in the same CA1 PNs, we verified that RyR-dependent Ca^2+^ release worked even in the absence of IP_3_R-dependent Ca^2+^ release from internal stores in Orai2−/− mice (Fig. 2c, d) as well as in WT mice when IP_3_-sensitive stores were depleted (Supplementary Fig. 5). In order to test the functional independence of IP_3_R- and RyR-dependent Ca^2+^ release, we designed an experiment in which we applied DHPG and caffeine sequentially to the same CA1 PNs. Fig. 2e, f demonstrates that incubation with Ry caused the well-known strong suppression of caffeine-evoked Ca^2+^ transients^10^, while DHPG-evoked Ca^2+^ transients were hardly affected at all. Together, the results presented in Fig. 2c–f and in Supplementary Fig. 5 indicate that IP_3_R- and RyR-dependent Ca^2+^ stores can release Ca^2+^ independently from each other.

**Fig. 3:**
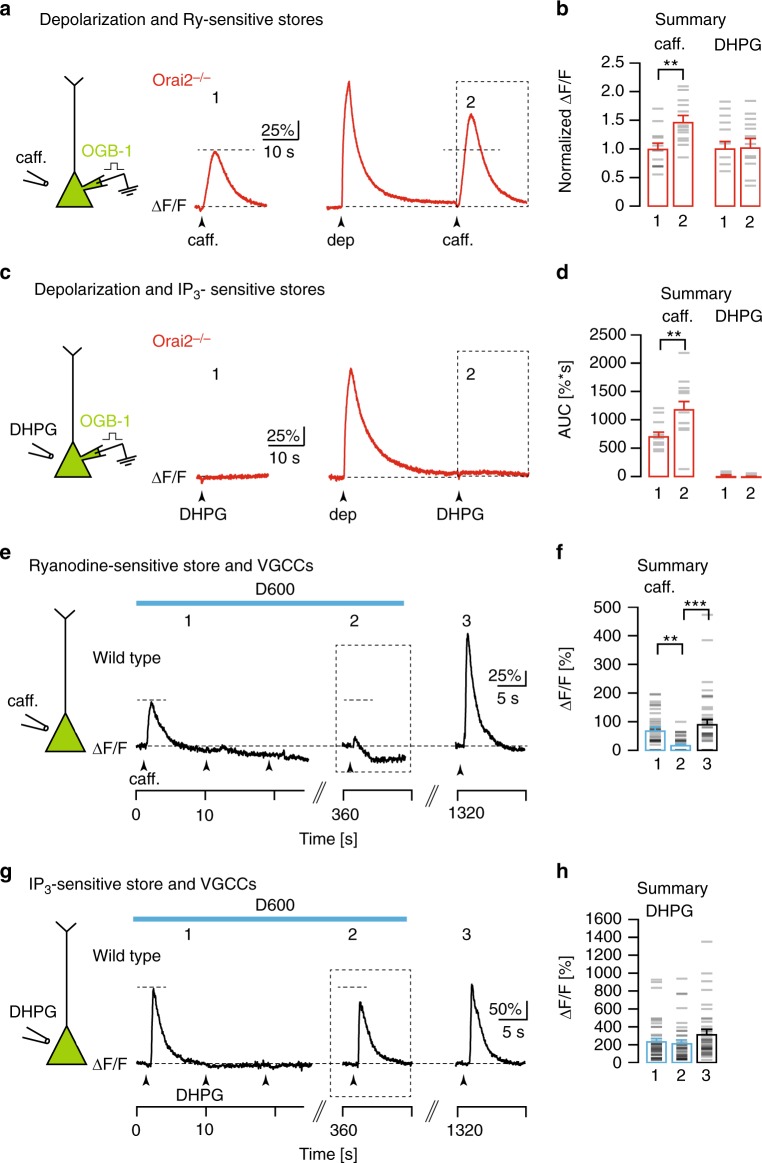
Orai2-independent replenishment and overcharging of Ry-sensitive Ca^2+^ stores. **a** Left: Caffeine was locally applied to the somata of whole-cell patch-clamped CA1 pyramidal neurons (PNs) filled with OGB-1 through the patch pipette. Red traces: Fluorescence recordings in an Orai2−/− mouse in response to applications of caffeine before (1), and 30s after (2) a depolarization to 0 mV for 1 s. **b** Summary of the experiments shown in **a** (left bars) and **c** (right bars). Bar graph showing mean amplitudes of relative fluorescence changes in response to caffeine (**a**, *n* = 13 cells, *p* = 0.0014) or 3,5-dihydroxyphenylglycine (DHPG; **c**, *n* = 17 cells, *p* = 0.65) application before (1) and after the depolarization (2), normalized to the control amplitude (1). **c** Analogous experiment in another CA1 PN from the same mouse with DHPG applied after the depolarization. **d** Mean area under the curve for 15 s following the caffeine (2) and DHPG (3) applications following the depolarization (marked by dashed boxes in **a** and **b**, caffeine: *p* = 0.01, DHPG: *p* = 0.831). **e** Left: Caffeine was locally applied to somata of CA1 PNs filled with Cal520-AM by multi-cell bolus loading (MCBL). Traces: Fluorescence recordings from the soma of a single CA1 PN in a wild-type mouse during consecutive caffeine applications (arrowheads) in the presence of D600 (blue bar) and after washout of D600. **f** Summary of the experiments shown in **e**. Mean amplitudes of relative fluorescence changes in response to caffeine applications at the time points indicated in **e** (*n* = 41 cells, analysis of variance (ANOVA): *p* = 0.001 (1 vs 2) and *p* = 4.98 × 10^−6^ (2 vs 3). **g** Analogous experiment to **e** with DHPG instead of caffeine applications. **h** Analogous summary to **f** for the experiments shown in **g** (*n* = 41 cells, ANOVA: *p* = 0.694 (1 vs 2) and *p* = 0.058 (2 vs 3)). ** - very significant (*p* <= 0.01), *** - extremely significant (*p* <= 0.0001)

The functional independence of IP_3_- and Ry-sensitive Ca^2+^ stores was strongly supported by two additional lines of experiments. In a first series of experiments illustrated in Fig. 3a–d, we tested the “overcharging” phenomenon of Ca^2+^ stores^10,11^. The results show that, 30 s following a depolarizing pulse, causing Ca^2+^ entry through VGCCs, there was a marked increase of the caffeine-evoked Ca^2+^ transient in a CA1 PN from an Orai2−/− mouse (Fig. 3a, c, d). By contrast, depolarization was ineffective when applying DHPG in Orai2−/− mice (Fig. 3b-d), although “overcharging” of IP_3_-sensitive Ca^2+^ stores was previously shown to work reliably in cerebellar Purkinje neurons of WT mice^11^. In a second series of experiments, we compared the role of VGCCs for IP_3_- and Ry-sensitive Ca^2+^ store refilling. In line with earlier observations^10^, we confirmed an antagonist of VGCCs, D600, prevented the replenishment of depleted RyR-dependent Ca^2+^ stores (Fig. 3e, f). D600 effectively abolished depolarization-evoked Ca^2+^ responses but not RyR-dependent Ca^2+^ release in CA1 PNs (Supplementary Fig. 6). At the same time, the spontaneous replenishment of depleted IP_3_-sensitive Ca^2+^ stores (Supplementary Fig. 7) was only marginally affected by the presence of D600 (Fig. 3g, h). In addition to D600, we also tested more specific Ca^2+^ channel antagonists and found that a cocktail of antagonists for L/PQ/T/R-type Ca^2+^ channels had a similar blocking effect of depolarization- and caffeine-induced Ca^2+^ transients (Supplementary Fig. 8). Thus, in contrast to IP_3_-sensitive stores that almost entirely rely on Orai2 for Ca^2+^ homeostasis, Ry-sensitive stores seem to require VGCCs for spontaneous refilling at resting membrane potential and can be overcharged by depolarization-induced Ca^2+^ influx through VGCCs.

## Discussion

In conclusion, several lines of evidence establish that IP_3_- and Ry-sensitive Ca^2+^ stores are functionally largely independent in hippocampal pyramidal neurons. First, IP_3_R-dependent Ca^2+^ release is functional in conditions in which Ry-sensitive Ca^2+^ stores are depleted or pharmacologically inactivated and vice versa for caffeine-dependent Ca^2+^ release. Second, Orai2 is selectively involved in the replenishment of IP_3_- but not Ry-sensitive Ca^2+^ stores. Third, by contrast, VGCCs are involved in the replenishment of Ry- but not IP_3_-sensitive Ca^2+^ stores. Fourth, depolarization-dependent overcharging of Ca^2+^ stores persists in Orai2−/− mice for caffeine-dependent but not for DHPG-dependent Ca^2+^ release from internal stores. Ry-sensitive stores that are functionally separate from IP_3_-sensitive stores have been previously reported in non-neuronal cells^22,23^ but not in central neurons^8^. However, the hypothesis of Ry and IP_3_ receptors sharing the same Ca^2+^ pool is solely based on IP_3_ uncaging experiments in cerebellar Purkinje^4^ without further molecular validation. Therefore, a first step in resolving the apparent discrepancy between hippocampal and Purkinje neurons may be an analysis of the role(s) of Orai channels in Purkinje neurons.

The functional independence of IP_3_- and Ry-sensitive Ca^2+^ stores in CA1 PNs is unexpected in view of the evidence that the ER forms a continuous network in neurons^24^. Possibly, the functional separation of the stores is the result of the clustering of IP_3_Rs and RyRs at sufficiently long distances within the ER network, with IP_3_Rs co-localizing with Stim molecules and Orai2, while RyRs co-localize with not yet identified types of VGCCs. Finally, our results help explain the distinct roles of IP_3_Rs and RyRs for various forms of long-term potentiation and long-term depression in hippocampal CA1 pyramidal cells^25–31^ and thus promote the development of specific molecular targets needed for a deeper analysis.

## Methods

### Mice

All experimental procedures were in compliance with institutional animal welfare guidelines and were approved by the state government of Bavaria, Germany. Mice were maintained in an animal facility under a 12-h light/dark cycle and food and water was provided ad libitum. All the animal experiments were performed in accordance with the policies established by institutional animal welfare guidelines of the government of Bavaria, Germany. Orai1^*loxP*/−^ and Orai1^CA1KO/−^ mice were generated by breeding mice with *loxP*-flanked exons 2 and 3 in the *Orai1* gene^32^ to mice with a heterozygous expression of *Cre* under the control of the αCaMKII-promoter (T29-1)^33^. *Cre/loxP* recombination in the resulting offspring generates two types of genotypes: a CA1-PN-specific (CA1KO)^33^ or a general deletion (−) of the *Orai1* gene (own finding). In the latter mouse line, we heterozygously preserved the *loxP*-flanked *Orai1* gene and thus generated Ora1^*loxP*/−^ mice. Orai1^CA1ko/−^ mice were created by using appropriate breeding steps and careful genotyping. The procedures for Orai2 gene targeting and the generation of Orai2-deficient mice (Orai2−/−) have been previously reported^20^.

The genotypes of Orai1^loxP/−^, Orai1^CA1ko/−^, T29-1 mice, and Orai2−/− mice were confirmed by PCR of tail tissue. The *loxP* sites on the Orai1 locus were confirmed by the following primer pairs: *Orai1loxP* forward: 5’-CAG CGT GCA TAA TAT ACC TAA CTC TAC CCG-3’ and *Orai1LoxP* reverse: 5’-GTA TTG ATG AGG AGA GCA AGC GTG AAT C-3’; product size: WT 220 bp, Orai1^CA1ko/−^: 360 bp. The heterozygous deletion of *Orai1* was verified by the use of *Orai1KO* forward: 5’-GGC TGG GAG ACA CTA ACT TCC TAA GG-3’ and *Orai1KO* reverse: 5’-CAT ATT GTG ACG GGA GGT TTG CAG-3’; product size: WT 1890 bp, Orai1KO: 400 bp. The genotype of T29-1 mice was verified by the following primers: *Cre* forward 5’-GCC GAA ATT GCC AGG ATC AG-3’ and *Cre* reverse 5’-AGC CAC CAG CTT GCA TGA TC-3’; product size: 420 bp. Orai2−/− mice were confirmed by the use of the following primer pair: (Orai2−/− forward: 5’-ACA CGA GTC TCT TCT GTC GG-3’ and reverse: 5’-CAT CTA CCT GCC CCT ATC-3’; product size: ca. 700 bp).

### mRNA extraction and quantitative RT-PCR

CA1 tissue was excised from the mouse brains at P18. Subsequently, tissue mRNA was extracted by the use of a RNA isolation kit, NucleoSpin RNA Plus (Machery-Nagel, Germany). Purified tissue RNA was incubated in gDNA Wipeout Buffer (QuantiTect; Qiagen) for 2 min at 42 °C to remove genomic DNA. RT was performed by the use of the QuantiTect® Reverse Transcription Kit (Qiagen, Germany). After RT, 5 ng of cDNA from each sample was collected for further qPCR analysis.

Single-cell qPCR was performed as previously described^18^. Briefly, CA1 pyramidal neurons were collected through a glass pipette with the resistance of 1–3 MΩ, which was filled with (in mM) 50 Tris-HCl (pH8.3), 75 KCl, 3 MgCl_2_, and 10 dithiothreitol (Promega, USA). After the tip of the pipette reached the membrane of an individual pyramidal neuron in CA1 region, negative pressure was applied to move the cell into the tip of the pipette. The pipette with cell inside was immediately transferred into an RNAse-free Eppendorf tube and frozen in liquid nitrogen and then placed at −80 °C for long-term storage for further RT reaction. For RT, cells were de-frozen at 0 °C and mixed with RT cell lysis buffer, which contained 2 mM dNTPs (Promega), 0.2% Igepal (Sigma), 40 U RNAsin® Plus RNAse Inhibitor (Promega, USA), 10 µM N6 random primers (Roche, Germany) at 70 °C for 5 min. After 5 min, 200 unit (U) of MMLV reverse transcriptase (Promega, USA) were added to start the RT reaction. RT was carried on at 37 °C for 2 h. A QIAEX II Gel Extraction Kit (Qiagen, Germany) was used for cDNA clean-up for the following LightCycler (Roche) qPCR reaction.

Five ng of tissue cDNA or 80% of the total extracted single-cell extracted cDNA per cell were mixed with the qPCR reaction solution “LightCycler Faststart DNA Master SYBR Green I” (Roche) and 0.5 µM of primer sets to amplify Orai1 (accession number NM 175423, forward: 5’-CCT GTG GCC TGG TTT TTA TC-3’ and reverse: 5’-GTG CCC GGT GTT AGA GAA TG-3’; product size: 160 bp), Orai2 (accession number AM712356, forward: 5’-ACC ATG AGT GCA GAG CTC AA-3 and reverse: 5’-GAG CTT CCT CCA GGA CAG TG-3’; product size: 162 bp), Orai3 (accession number NM 198424, forward: 5’-GGG TAA ACC AGC TCC TGT TG-3’ and reverse: 5’-GCC TGG TCC ATG AGC ACT AT-3’; product size: 166 bp), Itpr1 (accession number NM 010585, PrimerBank ID 6754390a1, forward: 5’-GGG TCC TGC TCC ACT TGA C-3’ and reverse: 5‘-CCA CAT CTT GGC TAG TAA CCA G-3’; product size: 144 bp), Itpr2 (accession number NM 019923, PrimerBank ID 26326875a1, forward: 5’-TTC AGT TCC TAT CGA GAG GAT GT-3’, and reverse: 5’-GCT GAT TGA CGC AAG GTC G-3’; product size: 140 bp), Itpr3 (accession number NM 080553, PrimerBank ID 162287074c2, forward: 5’-AAG TAC GGC AGC GTG ATT CAG-3’ and reverse: 5’-CAC GAC CAC ATT ATC CCC ATT G-3’; product size: 189 bp) and Gapdh (accession number BC 095932 forward: 5’-AGG TCG GTG TGA ACG GAT TT-3′ and reverse: 5´-TGT AGA CCA TGT AGT TGA GGT CA-3; product size: 141 bp). Itpr1, Itpr2, and Itpr3 primer sets are from PrimerBank^34^. Five ng of tissue cDNA or 10% of total harvested extraction was used for measuring the expression level of the house-keeping gene, Glyceraldehyde dehydrogenase (*Gapdh*) as internal control for later normalization. The relative expression level of each gene was measured by the following formula:

1 $$80\% \,{\mathrm{Expression}}\,{\mathrm{level}}_{(Orai)} = 2^{ - {\mathrm{Cp}}(Orai)}$$

2 $$10\% \,{\mathrm{Expression}}\,{\mathrm{level}}_{(Gapdh)} = 2^{ - {\mathrm{Cp}}(Gapdh)}$$

3 $${\mathrm{Normalized}}\,{\mathrm{expression}}\,{\mathrm{level}} = {\mathrm{Expression}}\,{\mathrm{level}}_{(Orai)}/{\mathrm{Expression}}\,{\mathrm{level}}_{(Gapdh)}$$

The crossing point (Cp) is calculated as the number of the reaction cycle in which the fluorescence intensity of SYBR Green I reaches a value above the noise level. In order to avoid genomic DNA contamination, samples that showed a positive signal without prior RT reaction were discarded from further analysis.

### Immunofluorescence staining

Mice were lethally anesthetized by isoflurane and then transcardially perfused with cold 4% paraformaldehyde solution (PFA; Merck). The brain was removed and post fixed with 4% PFA overnight at 4 °C. Then 70-μm sections were cut using a vibratome (Leica). For staining, slices were permeabilized for 15 min in phosphate-buffered solution (PBS), which contained 0.3% Triton-X-100 (Carlroth, Germany) at room temperature. Brain sections were blocked in PBS containing 10% fetal bovine serum (FBS; Gibco, USA) for 2 h at room temperature, followed by primary antibody hybridization overnight at 4 °C with chicken-anti-β-galactosidase antibody (1:100; Abcam) in 1% FBS. On the next day, slices were washed 10 min for three times in PBS containing 0.5% tween-20 (Sigma), and incubated with donkey-anti-IgY 649 Alexa Fluor (1:1000; Molecular probe) for 2 h at room temperature. After 10-min washing in PBS with 0.5% tween-20 for three times, brain slices were counterstained with DAPI (Sigma) to visualize cell nucleus and mounted by VECTASHIELD Antifade Mounting medium (Vector, USA). Images were acquired with a confocal laser scanning microscope (FLUOVIEW FV3000, Olympus; Japan). Images were processed with the ImageJ and Adobe Photoshop CS5 software (Adobe, USA).

### Acute hippocampal slice preparation

WT, Orai1^loxp/−^, Orai1^CA1ko/−^, and Orai2−/− mice on a C57BL/6 background at postnatal days P17 to P22 were used in the experiments. After the mice were deeply anesthetized with CO_2_ and decapitated, the brain was immediately removed and immersed in ice-cold slicing solution containing (in mM) 24.7 glucose, 2.48 KCl, 65.47 NaCl, 25.98 NaHCO_3_, 105 sucrose, 0.5 CaCl_2_, 7 MgCl_2_, 1.25 NaH_2_PO_4_, and 1.7 ascorbic acid (Fluka, Switzerland). The pH value was adjusted with to 7.4 with HCl and stabilized by bubbling with carbogen, which contained 95% O_2_ and 5% CO_2_, and the osmolality was 290–300 mOsm. Horizontal hippocampal slices 300 μm were cut in the slicing solution by the use of vibratome (VT1200S; Leica, Germany). Brain slices were kept in the recovering solution, which contained (in mM) 2 CaCl_2_, 12.5 glucose, 2.5 KCl, 2 MgCl_2_, 119 NaCl, 26 NaHCO_3_, 1.25 NaH_2_PO_4_, 2 thiourea (Sigma, Germany), 5 Na-ascorbate (Sigma), 3 Na-pyruvate (Sigma), and 1 glutathione monoethyl ester (Santa Cruz Biotechnology, USA) at room temperature for at least 1 h before the experiment. The pH value of the recovering solution was adjusted to 7.4 with HCl and constantly bubbled with carbogen, and the osmolality was 290 mOsm.

### Electrophysiological recordings

After resting in the recovery solution for at least 1 h, individual hippocampal slices were transferred to the recording chamber, which was constantly perfused at a flow rate of 2 ml/min with artificial cerebrospinal fluid (ACSF) containing (in mM) 2 CaCl_2_, 20 glucose, 4.5 KCl, 1 MgCl_2_, 125 NaCl, 26 NaHCO_3_, and 1.25 NaH_2_PO_4_ and gassed with 95% O_2_ and 5% CO_2_ to ensure oxygen saturation and to maintain a pH value of 7.4. In all, 30 μM D-AP5 (Abcam and Tocris, USA), 10 μm GYKI53655 (Tocris), and 10 μm bicuculline (Enzo, USA) were added to the ACSF to block NMDAR-, AMPAR-, and GABA_A_R-mediated synaptic transmission. The ACSF also contained 500 nM tetrodotoxin citrate (Abcam). Somatic whole-cell recordings from CA1 pyramidal neurons were performed with a borosilicate glass pipette with the resistance of ca. 7 MΩ filled with internal solution, which contained (in mM) 110 K-gluconate, 10 KCl, 10 HEPES, 4 Mg-ATP, 0.24 Na-GTP, 20 Na-Phosphocreatine (Sigma), and 100 μM of the fluorescent calcium indicator Oregon Green 488 BAPTA-1 hexapotassium salt (OGB-1; *K*_d_ 200 nM) (Molecular Probes, USA).The pH value of internal solution was adjusted to 7.3 with KOH. Voltage-clamp measurements were carried out using an EPC9/2 patch-clamp amplifier (HEKA, Germany). The membrane potential was held at −70 mV in voltage-clamp mode without liquid junction potential adjustment. Data acquisition and the generation of stimulation protocols were applied by the use of PULSE software (HEKA). Data were collected at 10 kHz and Bessel-filtered at 2.9 kHz and analyzed by the Igor 5 software (Wavemetrics, USA).

### Drug application

Depending on different purposes of experiments, the ACSF with antagonists against NMDARs, AMPARs, GABA_A_Rs, and voltage-gated sodium channels was used for bath application of the following drugs: cyclopiazonic acid (30 μM, Sigma), Ry (10 μM; Tocris), thapsigargin (5 μM, Sigma), D600 (500 μM, Sigma), nimodipine (20 μM ; Sigma), SNX-486 (0.5 μM; Tocris and Alomone), ω-Agatoxin IVA (0.2 μM; Peptide institute INC., Japan), and NiCl_2 _(75 μM; Sigma) as indicated in the respective “Results” section. Ca^2+^-free ACSF contained (in mM) 0.1 mM ethylene glycol-bis (β-aminoethyl ether)-*N*,*N*,*N*',*N*'-tetraacetic acid (EGTA; Sigma), 20 glucose, 4.5 KCl, 3 MgCl_2_, 125 NaCl, 26 NaHCO_3_, and 1.25 NaH_2_PO4. Local drug application was performed by a glass pipette with the resistance of 8 MΩ, which was connected to a Picospritzer II (Parker instrumentation, general valve, USA). The mGluR1/5 agonist DHPG (500 μM; Abcam) was dissolved in ACSF and Ry receptor agonist caffeine (40 mM, Sigma) was diluted in caffeine ringer, which contained (in mM) 2 CaCl_2_, 10 HEPES, 2.5 KCl, 1 MgCl_2_.6H_2_O, 120 NaCl, and 1.25 NaH_2_PO_4_. The pipette tip was placed at a distance of 15–20 μm from the cell soma.

### Calcium imaging

CA1 pyramidal neurons were dialyzed with an internal solution containing 100 μM of the calcium indicator OGB-1 after the whole-cell configuration was established. Alternatively, somata of pyramidal neurons were stained via multi-cell bolus loading (Stosiek et al.^35^) by pressure-application of 1 mM acetoxymethyl ester of the calcium-sensitive fluorescent dye Cal-520 (Cal-520-AM; *K*_d_: 320 nM, AAT-Bioquest, USA) in the staining solution using a Picospritzer. The preparation of the staining solution and the staining procedure were performed according to a standard protocol^35^. An upright multi-point confocal microscope (E600FN; Nikon, Japan) with a dual spinning disk (QLC 100; VTi, UK) was used for fluorescence image recording. A ×40 water-immersion objective with a numerical aperture (NA) 0.8 (Nikon, Japan) or a ×60 water-immersion objective with NA 0.9 (Nikon) were used for image acquisition. A 488-nm single wavelength Sapphire laser (Coherence, USA) was used for generating single-photon excitation of the Ca^2+^ indicator. The laser power under the objective was set to <0.15 mW. A CCD camera (NeuroCCD; RedShirt imaging, USA) with a resolution of 80 × 80 pixels was attached to the microscope for image acquisition. In order to synchronize the spinning disk and CCD camera during imaging, a function generator (TG1010A; TTi, UK) was used during recording. Image sequences were acquired by the use of Neuroplex software (RedShirt Imaging, USA) and analyzed with ImageJ with the help of the plug-in “Time Series Analyzer” (https://imagej.nih.gov/ij/plugins/time-series.html; NIH, USA). The fluorescence intensity in each somatic region of interest (ROI) (Supplementary Fig. 9) was corrected by background subtraction. A ROI immediately outside of the neuron was taken as background. Temporal fluorescence intensity changes in ROIs were expressed as relative percentage changes in fluorescence intensity: Δ*F*/*F* (%) = ((*F* − *F*_0_)/*F*_0_). *F*_0_ is defined as baseline fluorescence, which means the fluorescence intensity before a given stimulus, and *F* is the fluorescence change over time. Δ*F*/*F* (%) values were calculated and plotted using Igor Pro 5 (Wavemetrics, USA).

### IP_3_ uncaging

For photolytic release of IP_3_ experiments, 0.4 mM of NPE-IP_3_ (Invitrogen, USA) was supplemented in the internal solution. A tapered lensed optical fiber (working distance 6 ± 1 mm, spot diameter 6 ± 1 mm; Nanonics, Israel) was connected to a diode laser (Coherent Cube; 375 nm, 15 mW at the laser head) and placed on to the surface of the slice. Photolysis of caged IP_3_ was accomplished by a single light pulses for 50 ms through the optical fiber.

### Statistical analysis

Data were presented as mean ± SEM and plotted using Sigmaplot v.10 (Systat Software Inc., USA), unless noted otherwise. All statistical analysis was done with the use of the IBM SPSS statistics v.17 software (IBM, USA). Normal distribution was assessed with Shapiro–Wilk test. Statistical tests performed were two sided. For data sets with two independent experimental groups, independent Student’s *t* test was used for normally distributed data. For nonparametric (non-normally distributed) data, Mann–Whitney *U* test was used for statistical analysis. For two related experimental groups, paired Student’s *t* test was applied for normal distributed data or Wilcoxon matched-pairs signed-rank test for nonparametric data. To compare more than two experimental groups, one-way analysis of variance test [post hoc Least Significant Difference] was used. The linear fit and Pearson’s *r* in Fig. 1g were calculated using Sigmaplot v.10 (Systat Software Inc., USA).

### Reporting summary

Further information on experimental design is available in the Nature Research Reporting Summary linked to this article.

## Supplementary information

Supplementary Information

Reporting summary

Source data

## Data Availability

The data support the findings of this study (see Source Data) are available from the corresponding author upon reasonable request.
