# Supplementary material for: Two types of functionally distinct Ca2+ stores in hippocampal neurons
Source: Nat Commun. 2019 Jul 19;10:3223. doi: 10.1038/s41467-019-11207-8 (PMC6642203; doi:10.1038/s41467-019-11207-8)
Supplement: Supplementary file 1 — Supplementary Information [file 41467_2019_11207_MOESM1_ESM.pdf]

Two types of functionally distinct Ca^{2+} stores in hippocampal neurons

Chen-Engerer et al.

Supplementary Information

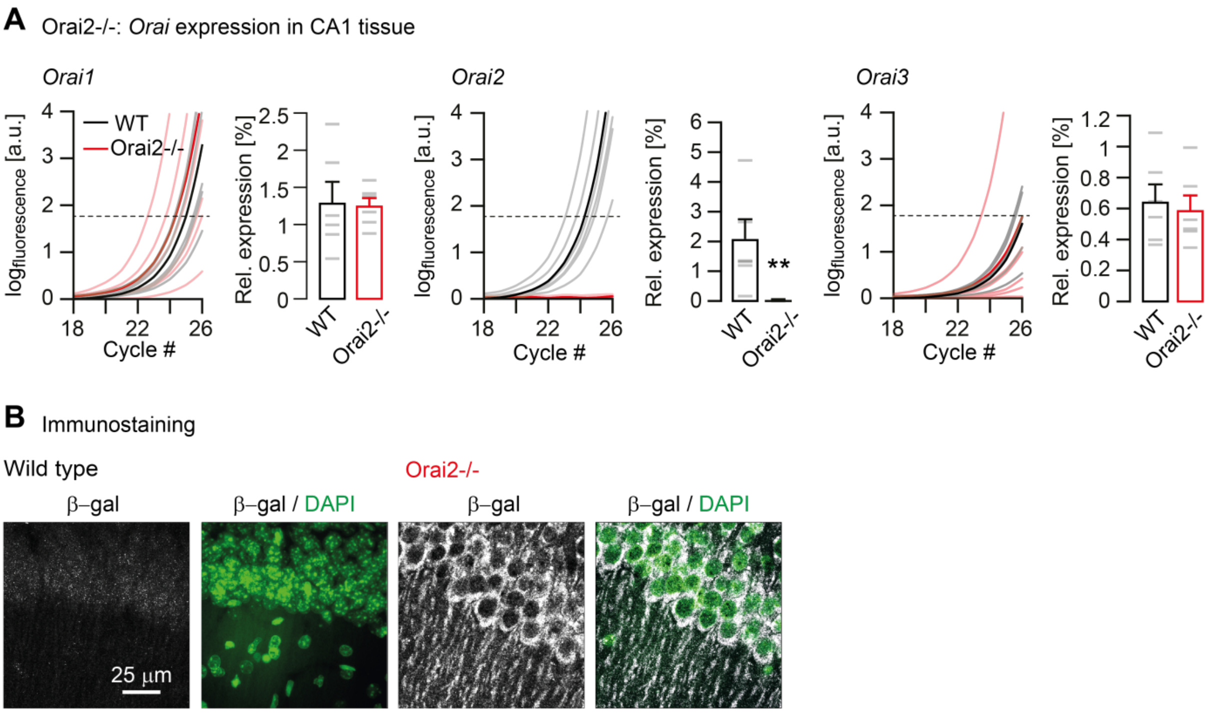

Supplementary Figure 1. Hippocampal expression of Orai genes in Orai2-/- mice.
a, Plots: Real-time monitoring of the fluorescence emission of SYBR Green I during the PCR amplification of Orai1-3 cDNA obtained by reverse transcription from mRNA harvested from CA1 tissue in Orai2-/- mice. Dashed lines: noise bands. Bar graphs: Mean expression levels of the three Orai homologs relative to the housekeeping gene Gapdh in CA1 tissue from Orai2-/- mice (Orai1: 1.25 ± 0.11%, Orai2: $6.35*10^{-3}$ ± $1.95*10^{-3}$%, Orai3: 0.58 ± 0.01% ; *n* = 6 mice). p-values for bar graphs from left to right: 0.699, 0.002, 0.699. **b,** Result of immunostaining for β-galactosidase (β-Gal) antibody (grey) with DAPI counterstaining (green) in the CA1 region in a wild type (left) and an Orai2-/- mouse (right; both at P18).

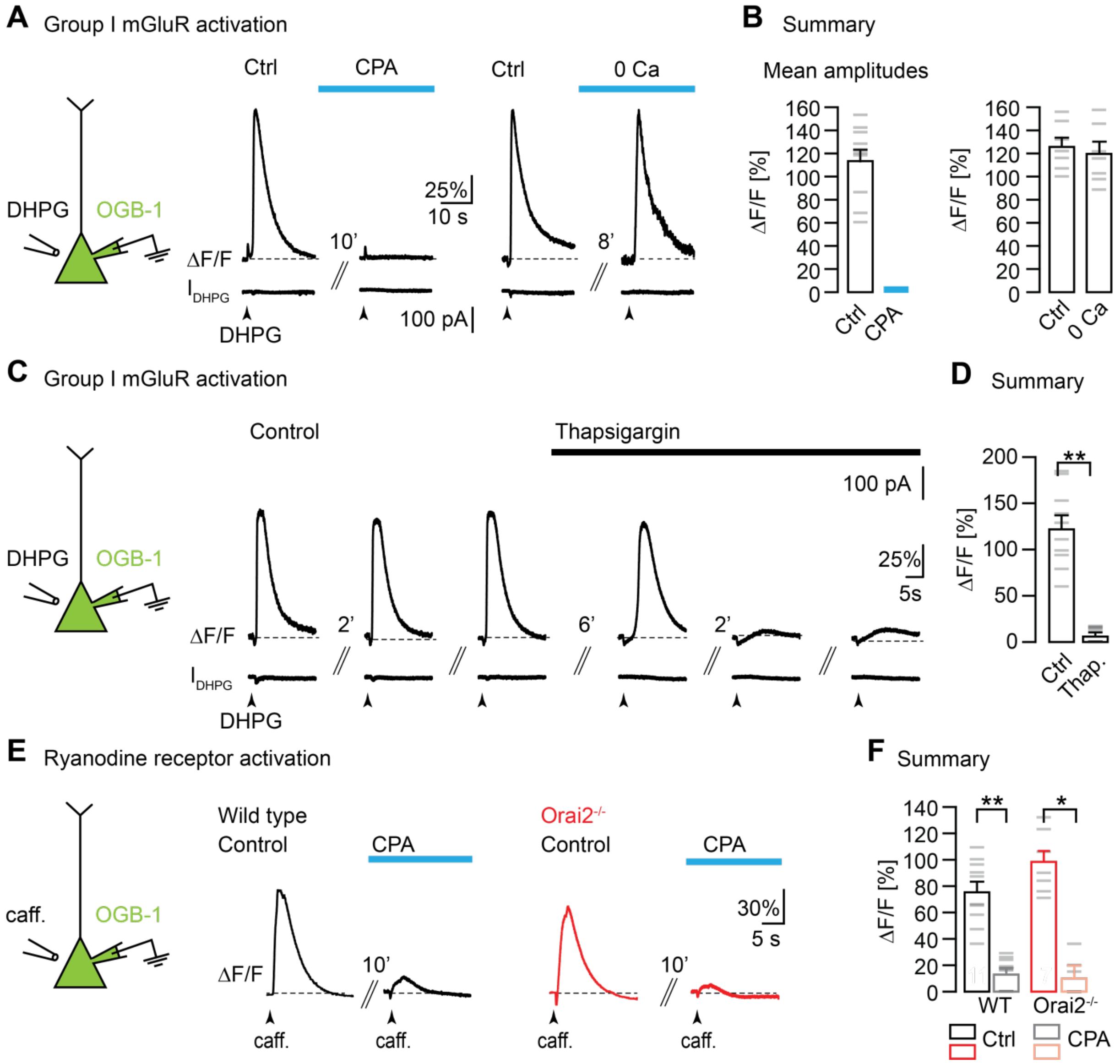

Supplementary Figure 2. Group I mGluR- and RyR-dependent Ca^{2+} release from ER Ca^{2+} stores
a, Left: DHPG was pressure-applied to somata of whole-cell patch-clamped CA1 PNs filled through the patch pipette with OGB-1. Left traces: Ca^{2+} transients and simultaneously captured voltage-clamp recordings in a CA1 PN in response to locally applied DHPG (500 µM, 200 ms) in control ACSF and in the presence of 30 µM CPA. Right traces: Analogous experiment in control ACSF and in nominally Ca^{2+} free extracellular perfusion media. Time intervals as indicated. **b,** Mean amplitudes of responses to DHGP application in control ACSF (113.6 ± 10.01 %) and in the presence of CPA (0; left; n=10 cells) and Ca^{2+} free external solution (control ACSF: 125.86 ± 7.83; 0 Ca: 119.86 ± 9.59; right; n=9 cells; p = 0.398), respectively. **c,** Left: DHPG was pressure-applied to somata of whole-cell patch-clamped CA1-PNs filled through the patch pipette with OGB-1. From left to right: First three traces show somatic fluorescence recordings and concomitant voltage-clamp recordings (bottom) in response to somatic DHPG applications (500 µM, 200 ms, 10 psi) with an interpulse interval of 2 min. When thapsigargin (3 µM) is washed in (black bar on top) the DHPG-evoked Ca^{2+} response persists for another 6 min but is abolished after that (last two traces). **d,** Mean amplitudes of relative fluorescence changes (ΔF/F) in (c)) (Control ACSF: 122.1 ± 13.33%, with thapsigargin:6.1 ± 2.5%, *n* =10 cells, p = 0.005). **e,** Left: Caffeine was pressure-applied to somata of whole-cell patch-clamped CA1-PNs filled through the patch pipette with OGB-1. Black traces: Fluorescence recordings with caffeine application in control

ACSF (left) and 10 min after starting the perfusion with CPA-containing external solution (blue bar, right). Red traces: Analogous experiment in an Orai2-/- mouse. **f,** Mean amplitudes of relative fluorescence changes ($\Delta F/F$) as in (e) (Wild type mice: 75.4 ± 7.7% in control ACSF and 13.1 ± 3.76% in the presence of CPA (*n* =10 cells, p = 0.005), Orai2-/- mice : 98.43 ± 8.89% and 10.14 ± 5.34%(*n* =7 cells, p = 0.018).

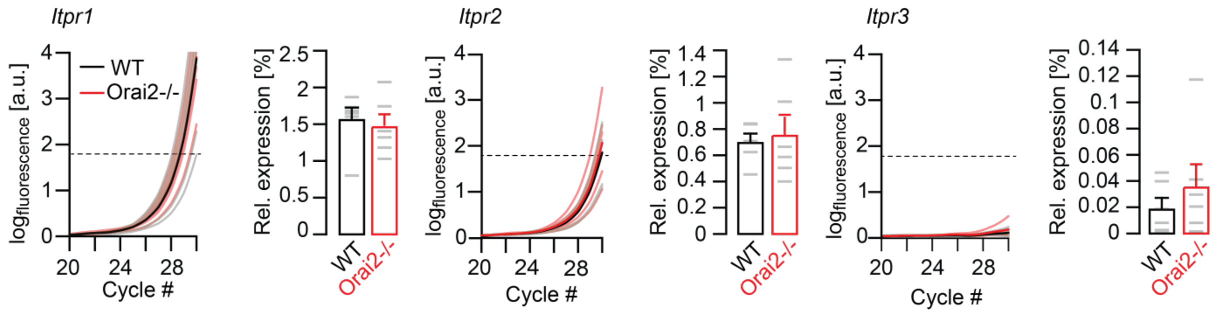

Supplementary Figure 3. Expression of *Itpr* genes in CA1 is not altered by the deletion of Orai2. *Plots*: Real-time monitoring of the fluorescence emission of SYBR Green I during the PCR amplification of *Itpr1-3* cDNA obtained by reverse transcription from mRNA harvested from CA1 regions excised from acute hippocampal slices as shown in Fig. 1A in wild type (*black*) and Orai2-/- mice (*red*). Dashed lines: noise bands. *Bar graphs*: Mean expression levels of the three *Itpr* homologs relative to the housekeeping gene *Gapdh* in CA1 from wild type (*Itpr1*: 1.56 ± 0.16%; *Itpr2*: 0.695 ± 0.06%; *Itpr3*: 0.018 ± 0.01%; n = 6 mice) and Orai2-/- mice (*Itpr1*: 1.46 ± 0.16%; *Itpr2*: 0.75 ± 0.14%, *Itpr3:* 0.035 ± 0.02%; n = 6 mice). p-values for the bar graphs from left to right: 0.699, 0.818, 0.699.

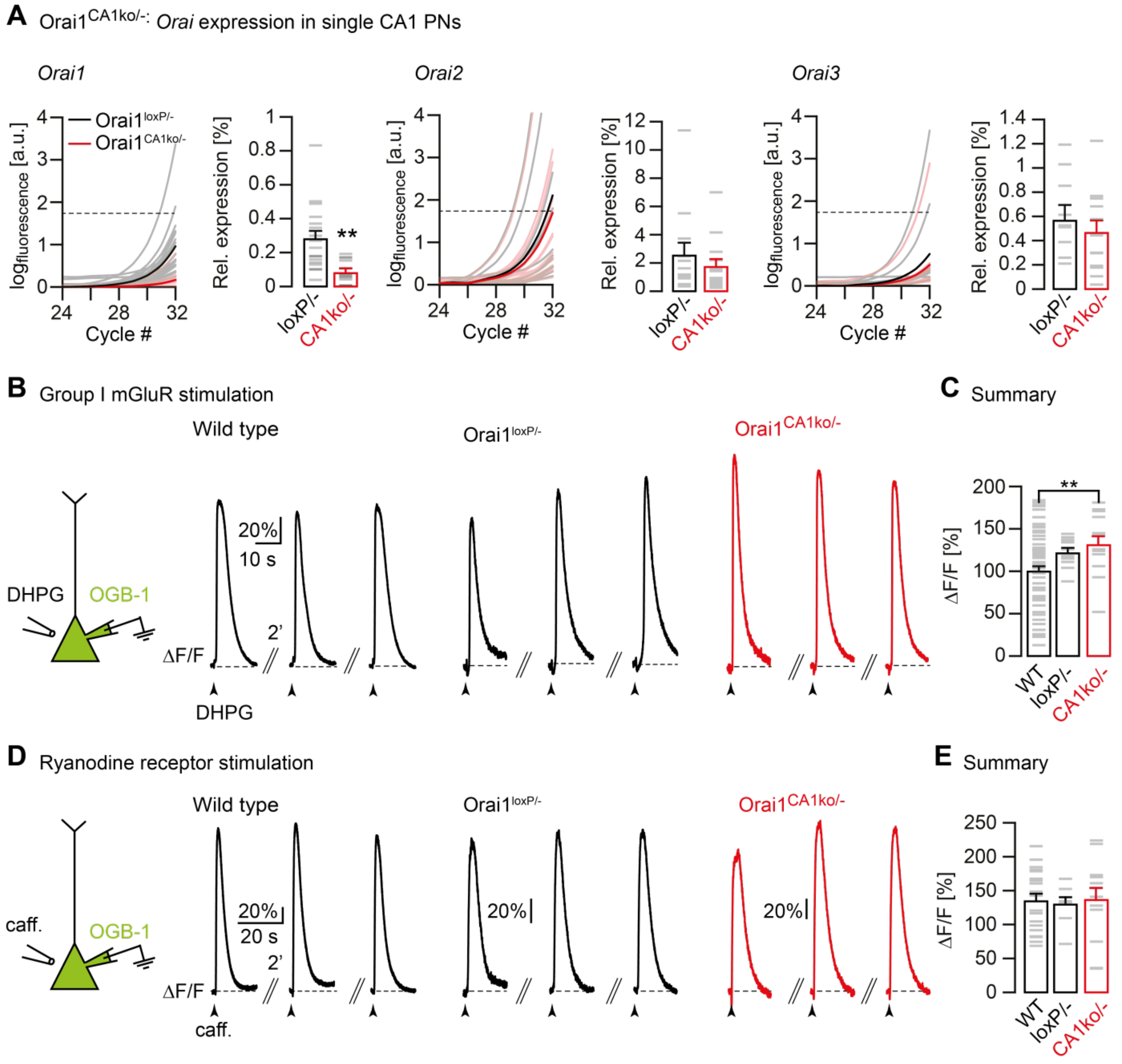

Supplementary Figure 4. Normal IP3R- and RyR-dependent Ca2+ release in the absence of Orai1

a, Plots: Real-time monitoring of the fluorescence emission of SYBR Green I during the PCR amplification of *Orai1-3* cDNA obtained by reverse transcription from mRNA harvested from single CA1 PNs in Orai1$^{CA1ko/-}$ mice. Dashed lines: noise bands. Bar graphs: Mean expression levels of the three *Orai* homologs relative to the housekeeping gene *Gapdh* in single CA1 PNs from Orai1$^{CA1ko/-}$ (*Orai1*: 0.08 ± 0.02%, *n* =13 cells; *Orai2*: 1.73 ± 0.49%, *n* =14 cells; *Orai3*: 0.46 ± 0.01%, *n* =12 cells) and Orai1$^{LoxP/-}$ mice (*Orai1*: 0.28 ± 0.05% *n* =17 cells; *Orai2*: 2.53 ± 0.84%, *n* =13 cells, *Orai3:* 0.57 ± 0.12%, *n* =10 cells). p-values for the bar graphs from left to right: $5.99*10^{-4}$, 0.458, 0.582. **b,** *Left*: DHPG was pressure-applied to somata of whole-cell patch-clamped CA1 PNs filled through the patch pipette with OGB-1. Black traces: Ca^{2+} transients in CA1 PNs in a wild type (left) and an Ora1$^{loxP/-}$ mice (right) in response to repeated local applications of DHPG (500 µM, 200 ms) at 2 min intervals. Red traces: Analogous experiment in an Orai1$^{CA1ko/-}$ mouse. **c,** Summary bar graph showing the mean amplitudes of DHPG-evoked Ca^{2+} transients in the three genotypes. (From left to right: *n* =62, 12, 14 cells). The mean amplitudes are 99.8 ± 5.9 % in the wild type, 121.3 ± 4.8 % in Orai1$^{loxP/-}$ mice and 131 ± 9.3 % in Orai1$^{CA1ko/-}$ mice (p = 0.014 (ANOVA)). **d,** Left: Caffeine was pressure-applied to somata of whole-cell patch-clamped CA1-PNs filled through the patch pipette with OGB-1. Black traces: Ca^{2+} transients in CA1-PNs in a

wild type (left) and an $Ora1^{loxP/-}$ mice (right) in response to repeated local applications of caffeine (40 mM, 3 s) at 2 min intervals. Red traces: Analogous experiments in an $Orai1^{CA1ko/-}$ mouse. **e,** Summary bar graph showing the mean amplitudes of caffeine-evoked Ca^{2+} transients in the three genotypes. (From left to right: 134.5 ± 9.98%, 129.62 ± 10.16%, 136.5 ± 17.9% ; *n* =20, 8, 12 cells; p = 0.91 (ANOVA)).

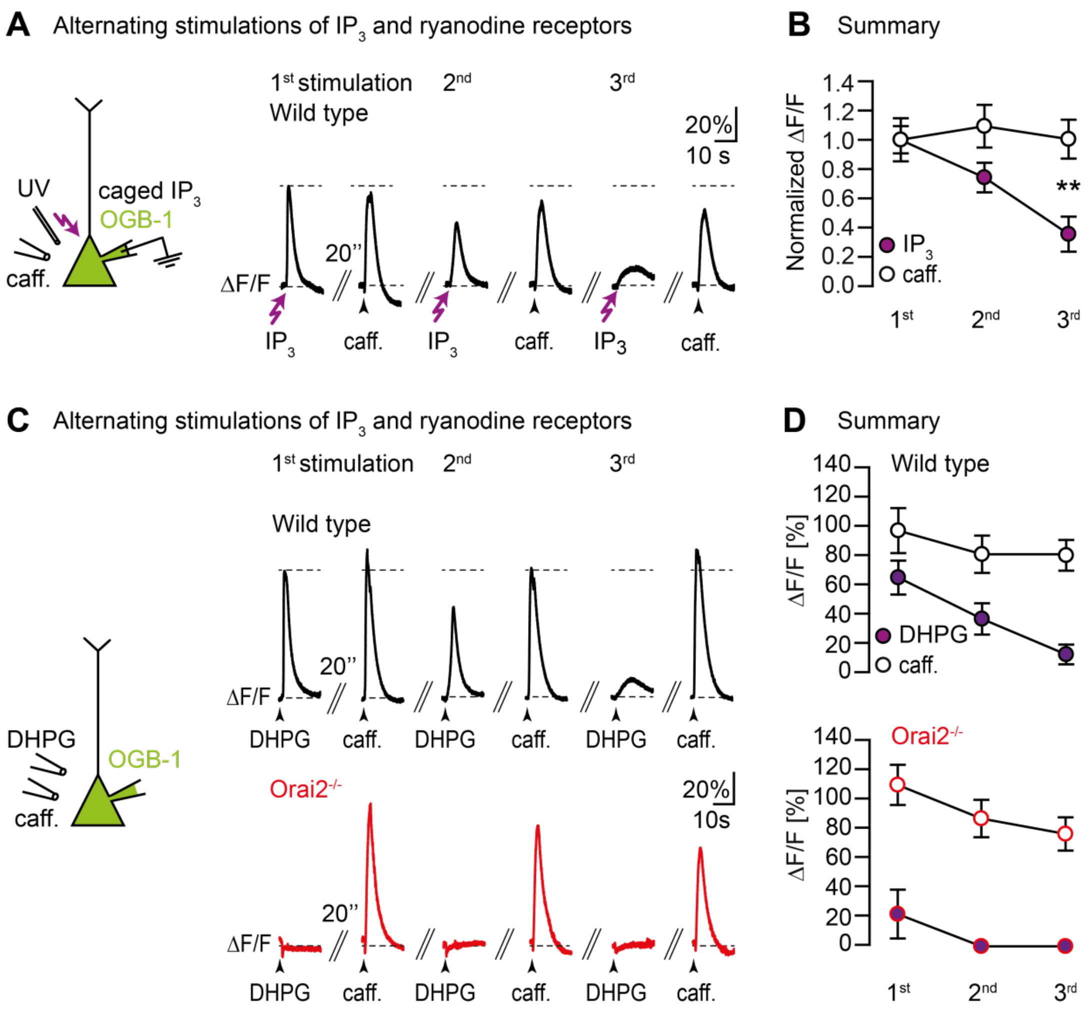

Supplementary Figure 5. Independence of IP_3- and Ry-sensitive Ca^{2+} stores.

a, Left: Both UV light pulses and local caffeine puffs were applied to somata of whole-cell patch-clamped CA1-PNs filled with OGB-1 and NPE ("caged")-IP_3 through the patch pipette. Traces: Fluorescence transients resulting from alternating photolysis of caged IP_3 and local DHPG puff applications at ISIs of 20'' as indicated in the same cell in a wild type mouse. **b,** Summary of the experiments as in (a). Mean amplitudes of relative fluorescence changes (ΔF/F) for the three stimulation circles with IP_3 (purple) and caffeine (white), normalized to the first amplitude. Mean third amplitude is 1 ± 0.13 for caffeine application and 0.36 ± 0.12 for IP_3 uncaging (*n* = 12 cells, p = 0.023). **c,** Left: Both caffeine and DHPG were locally applied to the somata of whole-cell patch-clamped CA1-PNs filled with OGB-1 through the patch pipette. Black traces, top: Fluorescence transients resulting from alternating local DHPG and caffeine puff applications at ISIs of 20'' as indicated in the same cell in a wild type mouse. Red traces, bottom: Analogous experiment in an Orai2-/- mouse. **d,** Summary of the experiments as in (c). Top: Mean amplitudes of relative fluorescence changes (ΔF/F) for the three stimulation circles with DHPG (purple) and caffeine (white), normalized to the first amplitude in the wild type. Mean third amplitude is 12.09 ± 6.75% for DHPG and 79.82 ± 10.49% for caffeine application, respectively (*n* = 11 cells). Bottom: Analogous summary for the experiments in Orai2-/- mice. Mean third amplitude is zero for DHPG and 77.93 ± 11.53% for caffeine application, respectively (*n* = 14 cells).

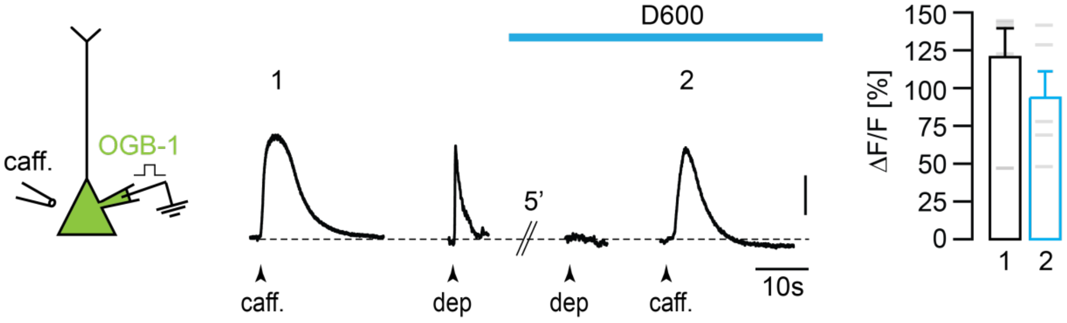

Supplementary Figure 6. Normal RyR-dependent Ca^{2+} release in the presence of D600.
Left: Caffeine was locally applied to the somata of whole-cell patch-clamped CA1 PNs filled with OGB-1 through the patch pipette. Traces, from left to right: OGB1 fluorescence in response to caffeine application (40 mM, 3s; 1), to somatic depolarization (100 ms, 0 mV) under control conditions and after 5 min of perfusion with 500 µM D600, and to caffeine application in the presence of D600 (2) in the same cell. Right: Bar graph showing the mean amplitudes of caffeine responses under control conditions (black, 1; $\Delta F/F$ =121.2 ± 18.7%) and in the presence of D600 (blue, 2; $\Delta F/F$ = 94.2 ± 18.1%; n = 5, p = 1.144, Wilcoxon signed ranks test). Scale bar: 50% for caffeine responses, 11% for responses to depolarization.

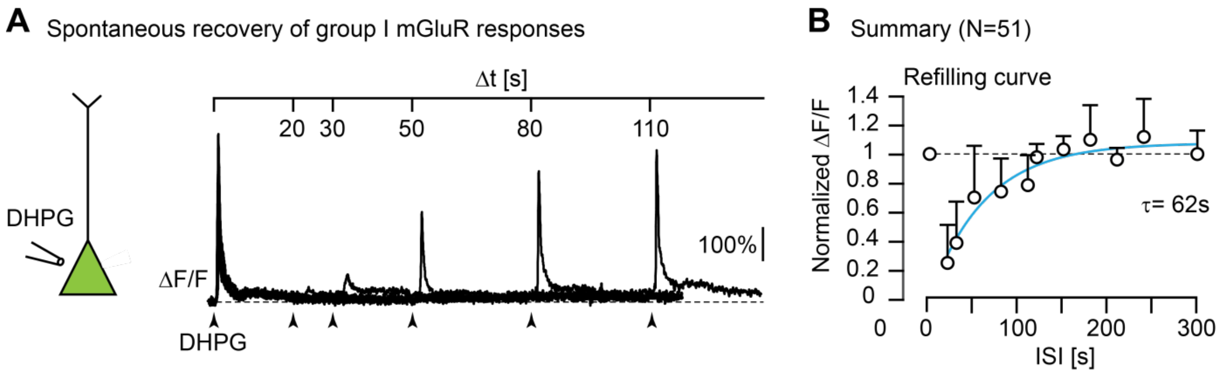

Supplementary Figure 7. Spontaneous refilling of IP_3-sensitive Ca^{2+} stores.

a, Left*:* DHPG was applied to somata of CA1 PNs filled with the Ca^{2+}-sensitive fluorescent dye Cal-520 AM by multi-cell bolus loading[1]. Right*:* Overlay of somatic fluorescence recordings from a single CA1 pyramidal neuron loaded with the Cal520-AM calcium indicator. Ca^{2+} transients were elicited by two paired DHPG pulses (500 ms, 500 µM, 10 psi) at different time intervals as indicated on the top. For the figure, five traces were aligned to the time point of the first stimulation. **b,** The amplitudes of the second responses in (a) and analogous experiments were normalized to the amplitudes of the first responses and plotted against the lengths of inter-pulse intervals. The increase of normalized second responses reflects spontaneous refilling of the DHPG/IP_3-sensitive Ca^{2+} store. A single exponential fit to the data points yielded a time constant (τ) of 62 s for the spontaneous refilling. (Fitting curve: f(x) =1.076(1-exp (-x/62.14)) with r^2=0.9334 (*n*=51 cells)).

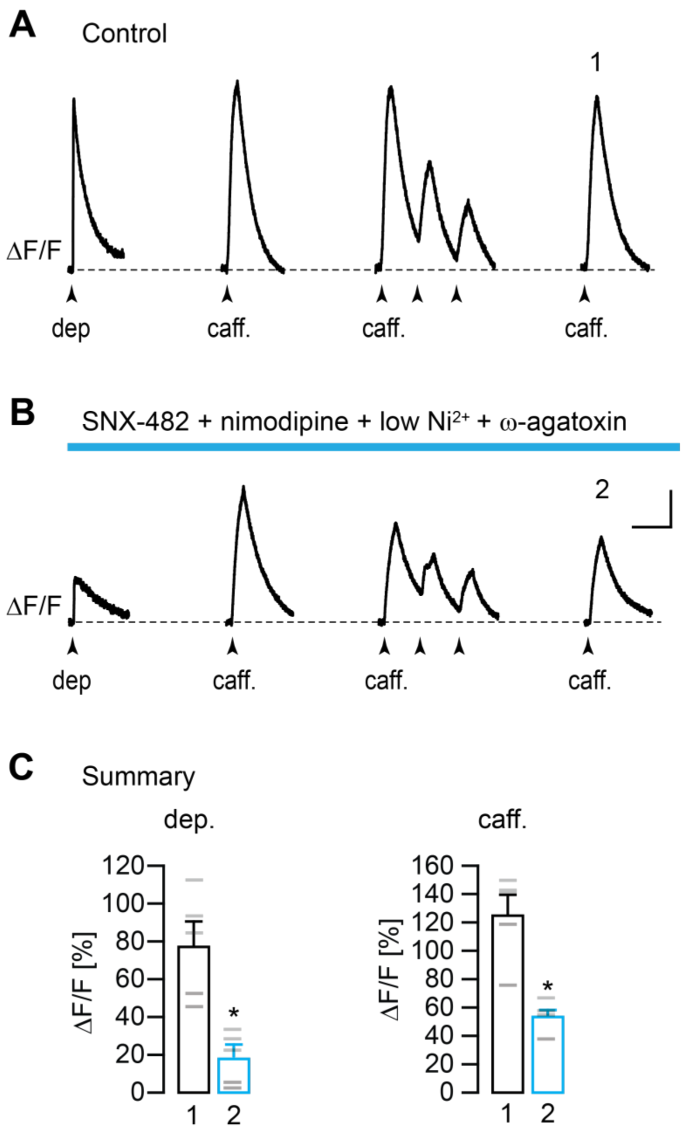

Supplementary Figure 8. Ry-sensitive stores and L/PQ/T/R-type VGCCs.

a, OGB1 fluorescence recordings from somata of whole-cell patch-clamped CA1 PNs in control ACSF. Traces from left to right: Response to depolarization (100 ms, 0 mV), response to single caffeine application (40 mM, 3s), first three out of six responses to repeated caffeine applications to deplete the Ry-sensitive store (ISI 2s), response to caffeine application after a recovery time of 2 min. **b,** Analogous responses from the same sequence of stimulations in the same cell in the presence of antagonists of VGCCs (R-type: 500 nM SNX-486; L-type: 20 µM nimodipine; T-type: 2 µM Ni^{2+}; PQ-type: 200 nM ω-agatoxin IVA). **c,** Summary of the experiments. Left: Bar graph showing mean amplitudes of Ca^{2+} transients resulting from depolarizations under control conditions (black; ΔF/F 77.2 ± 12.6%) and in the presence of VGCC-antagonists (blue; ΔF/F 18.0 ± 16.2%). Right: Analogous bar graph showing mean amplitudes of Ca^{2+} transients resulting from caffeine applications at time points 1 (ΔF/F 125 ± 13.5%and 2 (ΔF/F 53.6 ± 4.7%; n = 5; Wilcoxon signed ranks test) , respectively. Vertical scale bar: depolarization - 18%, caffeine – 20%. Horizontal scale bar: 10s.

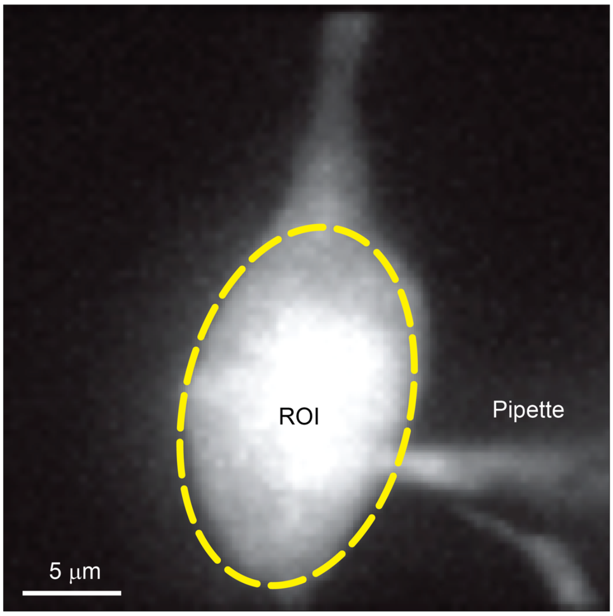

Supplementary Figure 9. Somatic regions of interest (ROIs) for analysis of fluorescence recordings.
Confocal image of a soma of a whole-cell patch-clamped CA1 PN filled with OGB1 through the patch pipette. The dashed yellow line encloses the region of interest (ROI) that was used for fluorescence measurements.
