# Supplementary material for: Two types of functionally distinct Ca2+ stores in hippocampal neurons
Source: Nat Commun. 2019 Jul 19;10:3223. doi: 10.1038/s41467-019-11207-8 (PMC6642203; doi:10.1038/s41467-019-11207-8)
Supplement: Supplementary file 2 — Reporting summary [file 41467_2019_11207_MOESM2_ESM.pdf]

nature research

Corresponding author(s): Arthur Konnerth

Last updated by author(s): May 3, 2019

Reporting Summary

Nature Research wishes to improve the reproducibility of the work that we publish. This form provides structure for consistency and transparency in reporting. For further information on Nature Research policies, see Authors & Referees and the Editorial Policy Checklist.

Statistics

For all statistical analyses, confirm that the following items are present in the figure legend, table legend, main text, or Methods section.

n/a	Confirmed	
☐	☒	The exact sample size (*n*) for each experimental group/condition, given as a discrete number and unit of measurement
☐	☒	A statement on whether measurements were taken from distinct samples or whether the same sample was measured repeatedly
☐	☒	The statistical test(s) used AND whether they are one- or two-sided<br>*Only common tests should be described solely by name; describe more complex techniques in the Methods section.*
☐	☒	A description of all covariates tested
☐	☒	A description of any assumptions or corrections, such as tests of normality and adjustment for multiple comparisons
☐	☒	A full description of the statistical parameters including central tendency (e.g. means) or other basic estimates (e.g. regression coefficient) AND variation (e.g. standard deviation) or associated estimates of uncertainty (e.g. confidence intervals)
☐	☒	For null hypothesis testing, the test statistic (e.g. *F*, *t*, *r*) with confidence intervals, effect sizes, degrees of freedom and *P* value noted<br>*Give P values as exact values whenever suitable.*
☒	☐	For Bayesian analysis, information on the choice of priors and Markov chain Monte Carlo settings
☒	☐	For hierarchical and complex designs, identification of the appropriate level for tests and full reporting of outcomes
☐	☒	Estimates of effect sizes (e.g. Cohen's *d*, Pearson's *r*), indicating how they were calculated

Our web collection on statistics for biologists contains articles on many of the points above.

Software and code

Policy information about availability of computer code

Data collection	Pulse v.8.8 (HEKA, Germany), Neuroplex v.10.2.2 (RedShirtImaging, USA), Olympus FV3IS-SW (Olympus, Japan), Lightcycler3 (Roche, Switzerland), Primer3 Input v.0.4.0 (open source, http://bioinfo.ut.ee/primer3-0.4.0/)
Data analysis	Igor v.5.0.1.0 (Wavemetrics, USA) with custom-made procedures, IBM SPSS statistics v.17 (IBM, USA), Sigmaplot v.10 (Systat Software Inc., USA), ImageJ (Fiji) v.1.52h with the Time Series Analyzer v.3 plugin (https://imagej.nih.gov/ij/plugins/time-series.html)

For manuscripts utilizing custom algorithms or software that are central to the research but not yet described in published literature, software must be made available to editors/reviewers. We strongly encourage code deposition in a community repository (e.g. GitHub). See the Nature Research guidelines for submitting code & software for further information.

Data

Policy information about availability of data

All manuscripts must include a data availability statement. This statement should provide the following information, where applicable:

- Accession codes, unique identifiers, or web links for publicly available datasets
- A list of figures that have associated raw data
- A description of any restrictions on data availability

The data that support the findings of this study are available from the corresponding author upon reasonable request.

Field-specific reporting

Please select the one below that is the best fit for your research. If you are not sure, read the appropriate sections before making your selection.

☒ Life sciences ☐ Behavioural & social sciences ☐ Ecological, evolutionary & environmental sciences

October 2018

Life sciences study design

All studies must disclose on these points even when the disclosure is negative.

Sample size	For each experiment, the sample size was determined based on literature in the field and prior experience with the applied analyses (e.g. Garaschuk et al., J. Physiol., 1997; Hartmann et al., Neuron, 2014). The precise number of mice or cells used are given in the text (main figures) or in the figure legends (supplementary).
Data exclusions	No data were excluded from the analysis.
Replication	Experiments were repeated so that our data are based on multiple independent repeats with similar results. The precise number of repeats are given in the text or figure legends (supplementary).
Randomization	Mice were allocated into experimental groups based on their respective genotypes (wild type, Orai1-deficient and Orai2-deficient).
Blinding	Investigators were not blinded to the genotypes of the mice.

Reporting for specific materials, systems and methods

We require information from authors about some types of materials, experimental systems and methods used in many studies. Here, indicate whether each material, system or method listed is relevant to your study. If you are not sure if a list item applies to your research, read the appropriate section before selecting a response.

Materials & experimental systems

n/a	Involved in the study	
☐	☒	Antibodies
☒	☐	Eukaryotic cell lines
☒	☐	Palaeontology
☐	☒	Animals and other organisms
☒	☐	Human research participants
☒	☐	Clinical data

Methods

n/a	Involved in the study	
☒	☐	ChIP-seq
☒	☐	Flow cytometry
☒	☐	MRI-based neuroimaging

Antibodies

Antibodies used	We used the polyclonal anti-beta-galactosidase antibody from Abcam, catalog number ab9361, lot number GR3192553-2.
Validation	The antibody was validated by the producer Abcam. In addition, the absence of staining in wild type mice demonstrates the specificity of the antibody.

Animals and other organisms

Policy information about studies involving animals; ARRIVE guidelines recommended for reporting animal research

Laboratory animals	We used mice (Mus musculus) of four different genocides: wild type (C57BL/6 strain), Orai1loxP/-, Orai1CA1ko/- and Orai2-/- (all mutants on a C57BL/6 background).
Wild animals	The study did not involve wild animals.
Field-collected samples	The study did not involve samples collected in the field.
Ethics oversight	All experimental procedures were in compliance with institutional animal welfare guidelines and were approved by the state government of Bavaria, Germany.

Note that full information on the approval of the study protocol must also be provided in the manuscript.
